# Allocation of Resources to Cyanogenic Glucosides Does Not Incur a Growth Sacrifice in *Sorghum bicolor* (L.) Moench

**DOI:** 10.3390/plants9121791

**Published:** 2020-12-17

**Authors:** Muhammad N. Sohail, Cecilia K. Blomstedt, Roslyn M. Gleadow

**Affiliations:** 1 School of Biological Sciences, Monash University, Wellington Rd, Clayton, VIC 3800, Australia; muhammad.sohail@sydney.edu.au (M.N.S.); Cecilia.Blomstedt@monash.edu (C.K.B.); 2 School of Life and Environmental Sciences, University of Sydney, Brownlow Hill, NSW 2570, Australia

**Keywords:** specialized metabolite, dhurrin, plant defence, seedling growth, phytohormones, cyanogenesis, resource allocation

## Abstract

In plants, the production of secondary metabolites is considered to be at the expense of primary growth. Sorghum produces a cyanogenic glycoside (dhurrin) that is believed to act as its chemical defence. Studies have shown that acyanogenic plants are smaller in size compared to the wildtype. This study aimed to investigate whether the small plant size is due to delayed germination or due to the lack of dhurrin derived nitrogen. A novel plant system consisting of *totally cyanide deficient class 1* (*tcd1*) and *adult cyanide deficient 1* (*acdc1*) mutant lines was employed. The data for germination, plant height and developmental stage during seedling development and final plant reproductive fitness was recorded. The possible role of phytohormones in recovering the wildtype phenotype, especially in developmentally acyanogenic *acdc1* line, was also investigated. The data on plant growth have shown that the lack of dhurrin is disadvantageous to growth, but only at the early developmental stage. The *tcd1* plants also took longer to mature probably due to delayed flowering. None of the tested hormones were able to recover the wildtype phenotype. We conclude that the generation of dhurrin is advantageous for plant growth, especially at critical growth stages like germinating seed by providing a ready source of reduced nitrogen.

## 1. Introduction

Secondary metabolites were originally believed to be the by-products of primary metabolism and considered not to be essential for plant survival [1]. However, recent research has shown that secondary metabolites promote plant fitness by improving plasticity under constantly changing biotic and abiotic environmental factors [2,3,4]. The most common types of secondary metabolites, or specialized metabolites as they are now known, are terpenes, phenolics and nitrogen or sulfur-containing compounds such as alkaloids, glucosinolates and cyanogenic glucosides [5]. They are generally not directly involved in primary metabolism, with some exceptions, such as the terpenes, sterols and carotenes [6]. Specialized metabolites are usually involved in a network of complex metabolic pathways, generally making it difficult to assign a specific function to them.

Cyanogenic glucosides (CNglcs) are nitrogen-based plant secondary metabolites present in more than 3000 species, representing angiosperms, gymnosperms and ferns [7]. CNglcs break down and release HCN after hydrolysis with a specific β-glucosidase [7,8]. This process, called cyanogenesis, is believed to have evolved for herbivore defence. However, there is growing evidence that cyanogenic glucosides are also involved in nitrogen turnover, transportation and in response to a variety of biotic and abiotic stresses [9,10]. Their role within the plant cell appears to depend on external environmental conditions. For example, under high availability of exogenous nitrogen the biosynthesis of CNglcs increases in plants [11,12,13], although this response is complex, and is dependent on other environmental factor, such as the degree of shade or stage of development [12,14]. Nevertheless, the induction of CNglcs in response to high nitrogen availability strongly suggests their possible involvement in nitrogen turnover and transportation, via an alternative turnover pathway. This pathway results in the formation of ammonia from CNglcs that can be used in plant growth and development, without the release of hydrogen cyanide (HCN) [15].

Sorghum produces the cyanogenic glucoside, dhurrin. When young, sorghum plants contain high concentrations of dhurrin, but this starts to decrease as the plant matures [11,16] consistent with the optimal allocation theory of plant defence [17]. In three-day old seedlings, dhurrin concentration can be as high as 6%, with high concentration of dhurrin localized in the growing shoot tips where it may constitute up to 30% of the total shoot dry weight [18]. Similar results were also reported by Adewusi [19] who showed that dhurrin concentration continued to increase in 5–7 day old seedlings, with much higher rates of synthesis in shoots than in the roots. Recently, it has been suggested that the role of dhurrin may not be limited to defence but also be involved in plant growth and development. The lack of dhurrin in mature seed and then the rapid biosynthesis within 24 h of imbibition is consistent with this although such results can also be ascribed to a purely defensive role [20,21].

Studies investigating the role of dhurrin in early growth and development of sorghum are scarce. A model system consisting of both cyanogenic and acyanogenic plants can provide a better system to investigate the role of CNglcs in a complex environment. Efforts to generate totally or partially acyanogenic plants have used RNAi or random mutagenesis and TILLING [8,22,23]. An acyanogenic mutant line, *totally cyanide deficient 1* (*tcd1*), lacks the ability to produce dhurrin due to a mutation in the key dhurrin biosynthesis gene, *CYP79A1* that prevents the enzyme from functioning [23]. Early observations of *tcd1* plants indicated that they are slow growing at the early seedling stage, but growth recovered at the adult stage [23]. This is similar to Jørgensen et al. [22] who found that acyanogenic cassava plants, created using RNAi, also displayed slower growth in the initial stages of seedling development. Another sorghum mutant, the *acdc1* line (*adult cyanide deficient class 1*), is cyanogenic at the seedling stage but becomes acyanogenic at later developmental stages [23]. Interestingly, this developmental mutant appears not to display slow growth in the days after germination stage, although this has not been well documented.

A number of studies hint at a link between turnover of CNglcs, germination and stress. Recently it has been proposed that during seed development dhurrin is endogenously turned over providing a store of reduced nitrogen and, at the same time, an increase in the biosynthesis of proanthocyanidins [20]. Proanthocyanidins may help regulate germination via their effect on the concentration of the hormone abscisic acid (ABA) [24,25]. In *Arabidopsis*, under conditions of low or no oxidative stress lack of proanthocyanidins promotes germination, but when oxidative stress is high, the absence of proanthocyanidins negatively affects the germination rate [25]. Proanthocyanidins are also known to increase ethylene synthesis [26]. HCN is produced as a co-product of ethylene biosynthesis [27], which in turn is known to promote germination in plants [28,29,30,31].

There is ongoing debate about whether CNglcs deployment comes at the cost of growth, as predicted by the various growth/defence trade-off hypotheses. If there is a trade off, then the hypothesis is that acyanogenic plants would have higher growth rates than their cyanogenic counterparts. The aim of this study was to find the reasons behind the slow growth of the acyanogenic mutant (*tcd1*) during early plant developmental stages. We hypothesized that this was either due to delayed germination or as a result of slow growth immediately after germination, as observed by Jørgensen et al. [22] for cassava. Gibberellic Acid (GA_3_) is a known stimulant for seed germination and subsequent plant growth and development [32,33]. If the *tcd1* plants are slow growing because of delayed germination, then exogenous GA_3_ application may recover the slow growth. To test this, the germination rate, plant height and stage of young seedlings were determined, as well as the effect of GA_3_ application on seed germination. Germination and growth were compared with the *acdc1* mutants, that have normal patterns of cyanogenesis in the first few weeks of growth, and with sibling lines that lacked the respective mutations (TCD1 and ACDC1) as well as the elite line from which the mutants were derived. The role of dhurrin in plant reproductive fitness was determined by analysing data for seed weight, days to flowering and final leaf stage at flowering. Plant hormones, in addition to GA_3_, were also applied exogenously to explore the possibility that the phenotype of the developmental mutant line (*acdc1*) was the result of a disruption in hormone signalling.

## 2. Results and Discussion

### 2.1. Role of Dhurrin in Seedling Development: Emergence, Plant Stage and Height

**Figure 1:**
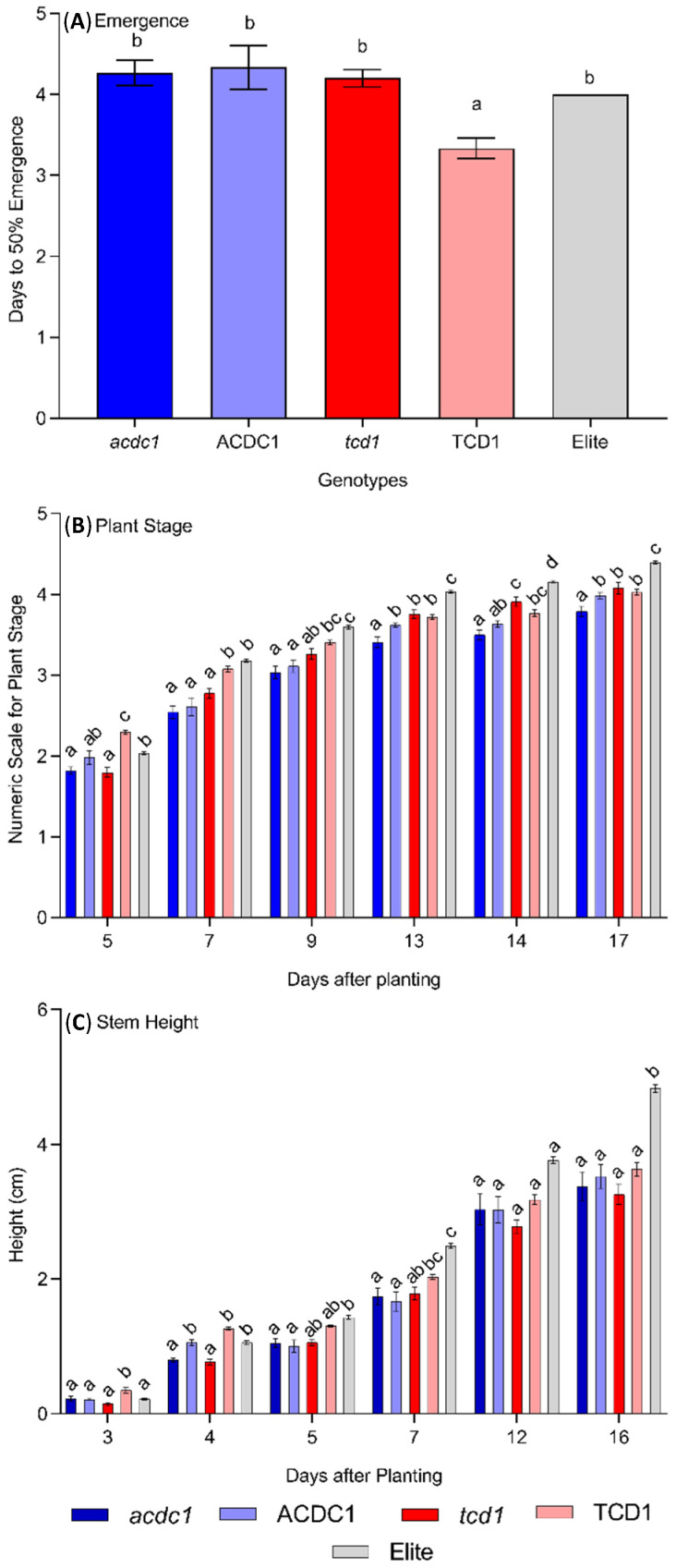
Seedling growth of sorghum genotypes (wild type parent, *adult cyanide deficient 1* (*acdc1)* mutant, *acdc1* sibling (ACDC1), *totally cyanide deficient class 1* (*tcd1*) mutant and *tcd1* sibling (TCD1)) recorded for 17 days after sowing in soil; (**A**) days to 50% emergence; (**B**) plant stage converted to a numeric scale; (**C**) plant height in cm. Each data point is mean of at least 250 replicates ± 1 standard error. Bars with the same letter at one timepoint are not significantly different from each other (*p* > 0.05; Tukey’s HSD pairwise test).

The first step in determining the effect of dhurrin on seedling growth and development was to measure the impact on emergence, stage and height during the first 17 days after planting (DAP) (Figure 1). Overall, during the earliest stages of development, all genotypes were at a similar developmental stage, but the two mutants were significantly shorter compared to the elite line (Figure 1B,C). This was not the result of differences in the rate of emergence with no significant difference detected between the rate of emergence of the mutants and the elite parental line (Figure 1A). Seeds from the nonmutated sibling line TCD1 emerged slightly earlier than any of the other genotypes but the reason for this is unclear. At 5 to 9 DAP *acdc1*, ACDC1 and *tcd1* plants were at similar plant stage, with both TCD1 and elite lines at a significantly more advanced developmental stage. However, a shift in this trend was observed from 13 DAP onward, where the elite line was at the most advanced stage, and all other genotypes were at a similar developmental stage, with the exception of *acdc1*.

A similar trend was observed in plant height, where a very sharp increase in height of the elite plants was observed from 7 DAP onwards (Figure 1C). At 3 DAP, the height of all genotypes except TCD1 was similar. TCD1 was the tallest with a height of 0.35 ± 0.04 (Figure 1C). At 4 DAP the height of elite, TCD1 and ACDC1 was significantly higher compared with the *tcd1* and *acdc1* mutants, with heights of 1.06 ± 0.03, 1.26 ± 0.02 and 1.06 ± 0.04, respectively, (Figure 1C). This is an important observation because TCD1 and ACDC1 would contain the same background mutations as the *tcd1* and *acdc1*, respectively, suggesting that the height difference is due to the specific mutations resulting in lower dhurrin content in these plants. After 5 DAP plants of the elite line remained significantly taller than the rest of the genotypes, at 7 (2.49 ± 0.04), 12 (3.76 ± 0.06) and 16 (4.83 ± 0.06) DAP (Figure 1C). Apparently, the lack of dhurrin in *tcd1* plants was only disadvantageous to growth at very early developmental stages (up until 7 DAP). From 7 DAP onwards the height of *tcd1* and *acdc1* remained similar to their respective sibling lines, TCD1 and ACDC1 (Figure 1C). At the end of this experiment, elite plants showed superior growth in both developmental stage and height, whereas the *acdc1* line remained behind only in plant development from rest of the mutant and sibling lines (Figure 1B).

Throughout the experiment the height of *tcd1* plants remained lower than the rest of the genotypes but remained statistically non-significant (Figure 1C). To check whether the slow growth of *tcd1* was due to a small embryo size, the embryo weight of elite, *tcd1* and TCD1 seeds were also measured (Table 1). The average embryo weight of TCD1 (0.42 mg) and *tcd1* (0.31 mg) was greater than the elite embryos (0.26 mg), suggesting the observed difference in growth was not due to an initial small embryo size. However, all the mutant and sibling lines were shorter compared to the elite line, and it is possible that this may be the result of random EMS-induced background mutations.

Blomstedt et al. [34] and Bjarnholt et al. [10] argue that the slow growth of *tcd1* plants may be the result of lack of reduced nitrogen derived from dhurrin. Data presented here are consistent with this view, but that this effect is only relevant at very early stages of development. During seed maturation dhurrin concentrations decrease until there is none present in mature seeds [20]. This decrease is due to the activity of the alternate turnover pathway as no dhurrinase transcripts were detected, suggesting that the detoxification pathway does not function in seeds. The turnover pathway would result in the storage of nitrogen in preparation for germination and growth. Therefore, the lack of dhurrin derived reduced nitrogen during seedling development could be the reason for slow growth of *tcd1* plants (Figure 1C). The data in the current study suggest that the positive effect of dhurrin on seedling growth is only visible at the early stage of plant growth and starts to disappear over time. This may be explained by the fact that at the start of germination in soil, the growing embryo mainly relies on seed nitrogen contents, but soon after emergence, the dependence for growth shifts from seed stored nutrients to photosynthesis. Another possible reason for shorter *tcd1* plants could be the lack of an auxin like compound, *p*-hydroxyphenylacetic acid [35]. This compound is derived from dhurrin turnover and may be acting as a weak auxin. Auxins are known to promote stem elongation in plants [36,37] and removing the dhurrin pathway may be reducing auxin-like activity [10,15,23].

### 2.2. Effect of Gibberellic Acid (GA_3_) on Germination and Early Plant Growth

**Figure 2:**
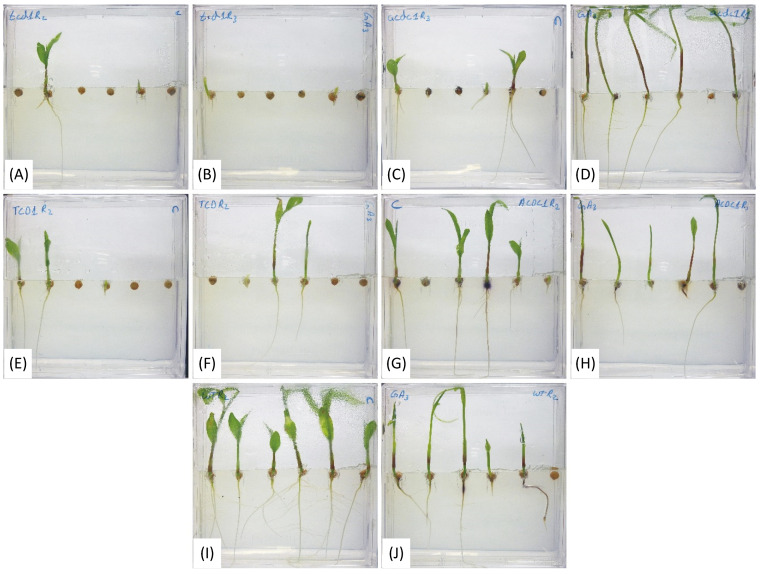
Effect of gibberellic acid (GA_3_) on five sorghum genotypes eight days after planting; (**A**,**B**) are *totally cyanide deficient class 1* (*tcd1*) mutants; (**C**,**D**) are *adult cyanide deficient 1* (*acdc1*) mutants; (**E**,**F**) are *tcd1* siblings (TCD1); (**G**,**H**) are *acdc1* siblings (ACDC1); (**I**,**J**) are elite; (**A**,**C**,**E**,**G**,**I**) are control treatment; (**B**,**D**,**F**,**H**,**J**) are GA_3_ treatment. A movie of the germinating seedlings can be found in Figure S1 and through the following link <https://youtu.be/fNWzvSFqchU>.

**Figure 3:**
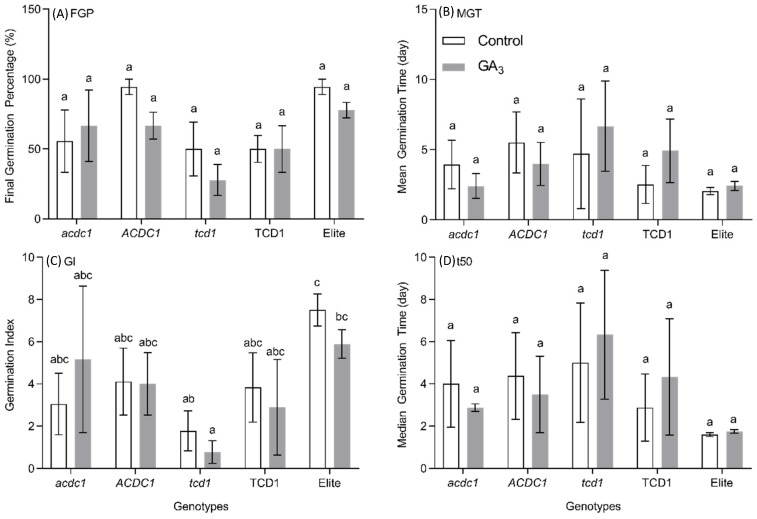
The effect of gibberellic acid (GA_3_) on germination of five sorghum genotypes: elite (parent line from which mutants are derived), *totally cyanide deficient class 1* (*tcd1*) mutant, sibling line of *tcd1* mutant without the target mutation (TCD1), *adult cyanide deficient 1* (*acdc1*) mutant and sibling line of *acdc1* mutant without the target mutation (ACDC1); (**A**): Final Germination Percentage (FGP); (**B**): Mean Germination Time in days (MGT); (**C**): Germination Index (GI); (**D**): Median Germination Time in days (t50). Bars with the same letter are not significantly different from each other (*p* > 0.05; Tukey’s HSD pairwise test).

**Figure 4:**
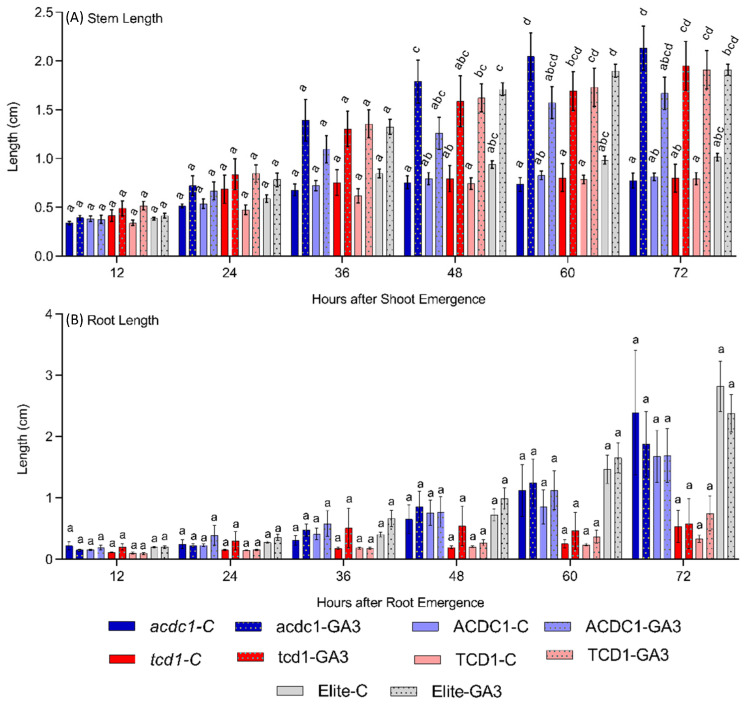
Effect of gibberellic acid (GA_3_) on early plant growth (root and stem length), of five sorghum genotypes: Elite (parent line from which mutants are derived), *totally cyanide deficient class 1* (*tcd1*) mutant, sibling line of *tcd1* mutant without the target mutation (TCD1), *adult cyanide deficient 1* (*acdc1*) mutant and sibling line of *acdc1* mutant without the target mutation (ACDC1); data were collected at 12 h intervals up until five days; (**A**) shoot length under control and GA_3_ treatment; (**B**) root length under control and GA_3_ treatment. Bars with the same letter at one timepoint, are not significantly different from each other (*p* > 0.05; Tukey’s HSD pairwise test).

The first experiment in this study examined the possible differences in rates of emergence of seeds from acyanogenic and cyanogenic plants (Figure 2). In order to capture more detail of the impact on germination itself, the same genotypes were grown in vitro on vertical plates and monitored for five days (Figure 2). A time-lapse video was made using images taken during germination of seeds in vertical plates, which can be found at <https://youtu.be/fNWzvSFqchU>. The data for plant germination were collected at 12 h intervals, up to 5 DAP (Figure 3). In addition to germination, root and stem length was also recorded up to 72 h after planting (Figure 4). The hormone GA_3_ plays important roles in both germination and plant growth. To investigate the potential effect of GA_3_ on the acyanogenic sorghum mutants, that we had already established were somewhat smaller, we grew all five genotypes under both control and GA_3_ treatments (Figure 2). While the germination indices showed high variability in the mutant and respective sibling lines, compared to the elite (Figure 3), there were several noteworthy observations. First, the final germination percentage (FGP) and mean germination time (MGT) values were not significantly different across all tested genotypes under either control or GA_3_ treatment (Figure 3A,B). The elite line showed maximum percentage of germination when grown in vitro (on MS medium in constant room temperature) (Figure 3A). Second, the *tcd1* mutant line showed lower FGP (27.78 ± 11.11) compared to the elite line (77.78 ± 5.56) under GA_3_ treatment (Figure 3A) although this was not statistically significant. In addition to low FGP, *tcd1* also took longer to germinate as indicated by a higher MGT (6.67 ± 1.86) value as compared to the elite (2.42 ± 0.19) under GA_3_ treatment (Figure 3B). Third, the germination index (GI), considered by some to be a better indicator of germination rate, was significantly lower in *tcd1* than the elite plants in both treatments (Figure 3C).

Plants growing in the GA_3_ treatment increased the internode length but did not affect the root length (Figure 2 and Figure 4), consistent with known functions of GA_3_ on growth. The difference in internode length increased over time, becoming statistically significant 60 h after shoot emergence for most of the genotypes (Figure 4A). Internode length did not vary across tested genotypes in either control or treated plants (Figure 4A). Exogenous GA_3_ application has been shown to be able to rescue the wild type phenotype in mutants lacking the ability to synthesize endogenous GA_3_ [38].

Overall stem length of all five genotypes was not significantly different in the control or GA_3_ treatment groups (Figure 4A). Thus, the hypothesis that the slow growth of *tcd1* might be due to lower endogenous GA_3_ is rejected. Another possibility is that that inability of *tcd1* to complete glycosylation of the aglycone could be causing an over accumulation of free glucose in the cytosol, altering sugar signalling and the downregulation of photosynthesis [39].

The observation that 72 h after germination both *tcd1* and elite lines are the same height is at odds with our observations in a previous experiment where *tcd1* plants were smaller than TCD1 and elite genotype at 3 DAP. An important difference between these two experiments is the availability of light. In the present experiment, seeds were grown in transparent medium and soon after the shoot emergence the growing seed can start to photosynthesise, whereas in the previous experiment, seeds were sown into soil, and were still below ground at this stage. A germinating plant is dependent on the energy and nitrogen resources in the seed until it can photosynthesise, on the one hand, and develop roots to access nutrients from the soil, on the other. We hypothesise that dhurrin may supply both reduced nitrogen and glucose in these early stages, and the lack of dhurrin in the *tcd1* mutants might be the reason for relatively slow growth of *tcd1* plants in soil. Whether this is a direct result of resource limitation or due to an imbalance in the carbon to nitrogen ratio, as proposed by Paul and Driscoll [40], cannot be determined from the results presented here.

### 2.3. Role of Phytohormones in Dhurrin Biosynthesis

**Figure 5:**
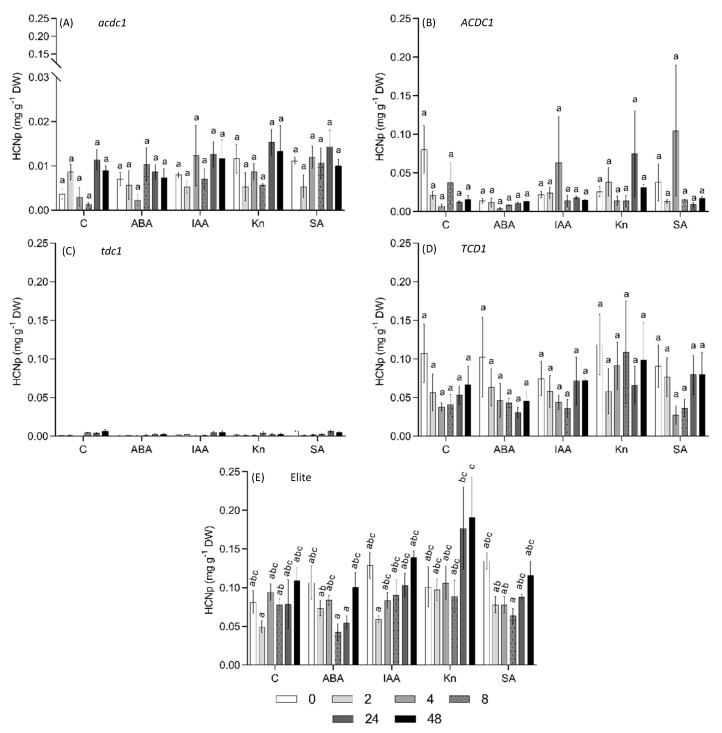
Cyanide potential (HCNp) of 18-day-old *S. bicolor* after foliar application of ABA (abscisic acid, 150 µM); IAA (indole-3-acetic acid, 100 µM); Kn (kinetin, 20 µM); SA (salicylic acid, 1 mM) and Control. (**A**) sibling line of *acdc1* mutant without the target mutation (ACDC1); (**B**) *adult cyanide deficient 1* (*acdc1*) mutant; (**C**) elite (parent line from which mutants are derived); (**D**) sibling line of *tcd1* mutant without the target mutation (TCD1); (**E**) *totally cyanide deficient class 1* (*tcd1*) mutant. Bars with the same letter are not significantly different from each other (*p* > 0.05; Tukey’s HSD pairwise test).

**Figure 6:**
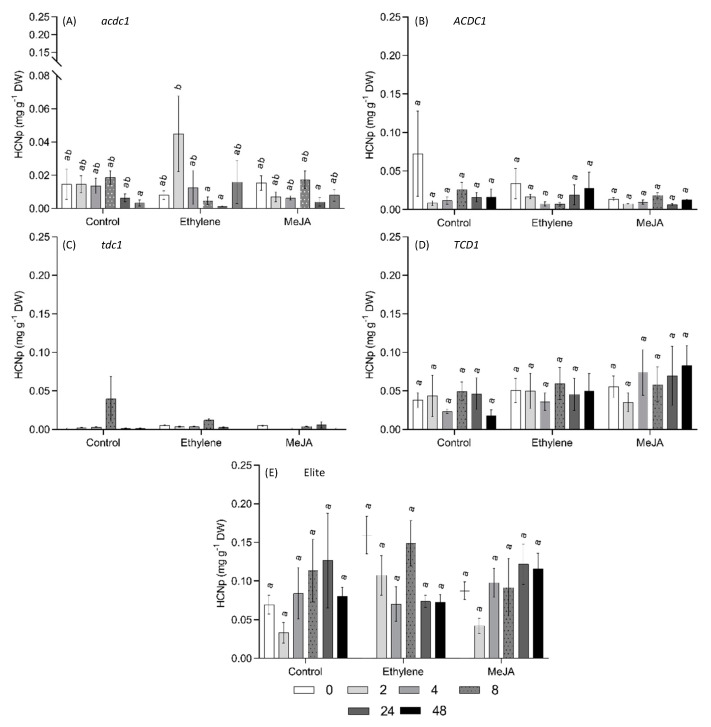
Cyanide potential (HCNp) of 18-day-old *S. bicolor* after foliar application of ethylene (1 mM); MeJa (methyl jasmonate, 100 µM) and Control. (**A**) elite (parent line from which mutants are derived); (**B**) sibling line of *tcd1* mutant without the target mutation (TCD1); (**C**) *totally cyanide deficient class 1* (*tcd1*) mutant; (**D**) *adult cyanide deficient 1* (*acdc1*) mutant; (**E**) sibling line of *acdc1* mutant without the target mutation (ACDC1); Bars with the same letter are not significantly different from each other (*p* > 0.05; Tukey’s HSD pairwise test).

The *acdc1* line used in this study has a mutation in the promoter of *CYP79A1* gene, the first step in the dhurrin biosynthesis pathway [41]. Based on the observation that this line is developmentally acyanogenic, i.e., dhurrin concentration decreased earlier in development and to a lower level compared with wild type plants, and that the mutation appears to be regulatory we hypothesized that this phenotype might be the result of altered phytohormone signalling. To investigate whether the *acdc1* phenotype is associated with a disruption in plant hormone biosynthesis or a signalling pathway, six different hormones were exogenously applied to young sorghum plants. In this experiment, the *tcd1* mutant line acts as a negative control of dhurrin synthesis. Overall, none of the applied hormones significantly changed the HCNp across all tested genotypes (Figure 5 and Figure 6). We conclude, therefore, that the altered pattern of dhurrin synthesis in the *acdc1* mutant is not hormonally mediated.

Drought is known to induce dhurrin synthesis in sorghum, and ABA is known for regulating the plant response to osmotic stress [42,43,44,45]. Jasmonate-responsive transcription factors are potential regulators of plant secondary metabolites in response to biotic stress [46], but relatively little is known about their effect on cyanogenesis [47,48]. In the present study, foliar application of ABA and MeJA did not result in any detectable change in the dhurrin synthesis in any of the tested genotypes, contrary to our hypothesis (Figure 5 and Figure 6). This could be because of the age of the plants being tested. We applied the hormones to very young plants (18 DAP) compared with 45 DAP in the study by Shehab et al [43]. Dhurrin concentrations are very high in young plants [14] and the genes are already highly expressed, thus any increase in expression may not be detectable. A more detailed experiment focusing on the effect of exogenous ABA and MeJA application on sorghum across different developmental stages might shed more light on the role of these hormones in dhurrin regulation.

### 2.4. Role of Dhurrin in Reproductive Fitness

Elite, *tcd1* and sibling TCD1 genotypes were grown to maturity in the glasshouse and data for days to flowering, seed weight and final leaf number were recorded (Table 1). Elite plants flowered 91 ± 1 DAP, significantly faster than TCD1 (97 ± 2 DAP) and *tcd1* (103 ± 1 DAP). Total leaf number at flowering was similar in both elite (12.4 ± 0.1) and *tcd1* (12.4 ± 0.3) genotypes (Table 1), thus while there is a difference in time to flowering there was no significant difference in developmental stage. The lack of dhurrin in *tcd1* might have caused the delay in flowering, hence requires more time to mature (Table 1). Lack of dhurrin was not associated with reduced seed weight, with the mass of *tcd1* seeds (19.8 ± 1.3) higher than the elite (18.0 ± 0.3) genotype (Table 1). At early stages of sorghum seed development dhurrin is present but begins to disappear as the seed matures, hence the mature seeds are acyanogenic [20]. The presence of dhurrin in immature seeds suggests it may play a role in reproductive development or as stored nitrogen. Other studies have also linked the presence of dhurrin at flowering with drought tolerance although not with changes in rate of development [49].

## 3. Implications for Function of Cyanogenesis in Defence and Growth

Slow growth, or the smaller size, of *tcd1* sorghum plants in the first few weeks of growth is not from delayed germination or sensitivity to GA_3_. It is more likely that the lack of readily available reduced nitrogen associated with rapid dhurrin synthesis after imbibition may be the reason why *tcd1* plants are shorter, and hence appear to be slow growing. The totally acyanogenic *tcd1* plants also take longer to mature and have delayed flowering. Dhurrin is believed to be primarily involved in plant defence and high concentration of dhurrin at germination may have evolved to prepare plants against possible herbivore attack. Resource allocation theories universally predict that diverting carbon and nitrogen to defence should result in a growth penalty. The fact that this has been hard to detect is generally attributed to the low concentrations of resources required to make these compounds, the low cost of synthesis, or that the costs of synthesis are offset by other beneficial processes. Here we demonstrate, using sorghum mutants lacking dhurrin, that dhurrin is advantageous for growth in the early stages of plant development, especially in the absence of photosynthesis, but not in older plants. Rather than being a cost, we speculate that the putative role of dhurrin as a source of nitrogen for the germinating seedlings is critically important for rapid growth of sorghum and may help it to establish in the dry tropics where rainfall may be unreliable.

## 4. Materials and Methods

### 4.1. Plant Material and Growth Conditions

Five *Sorghum bicolor* genotypes were used in this study: The parent line from which the mutants were derived (elite), the *adult cyanide deficient class 1* (*acdc1*), the *total cyanide deficient 1* (*tcd1*) mutants and two sibling lines from the mutated populations that are from the same initial selection but lack the respective mutation (ACDC1 and TCD1) [23]. Plants were grown in the Plant Science Complex, Monash University under natural light conditions with a mean temperature of 26.2 °C ± 7.2 day/19.5 °C ± 4.7 night, with a relative humidity of 51.7% ± 13.6/59.11% ± 11.9 day/night. Seeds were planted in Debco seed raising mix and perlite (3:1 ratio) in 135 mm pots (1.5 L capacity) pots or 30-cell seedling trays.

### 4.2. The Role of Dhurrin in Early Growth and Development of Sorghum

To investigate the role of dhurrin in early sorghum growth and development, 450 seeds of each genotype except *tcd1*, were grown in the greenhouse. For *tcd1* genotype 900 plants were grown instead of 450, because previous studies had shown that *tcd1* does not germinate well. The time taken for 50% of the seeds to germinate/emerge was recorded. Data for plant stage (leaf number) and stem height were measured for 17 days after planting (DAP). The data for plant stage were measured on 5, 7, 9, 13, 14, and 17 DAP. For plant height, data were recorded on 3, 4, 5, 7, 12, and 16 DAP. The data for plant stage were converted into numeric form and based on numerical rankings (Supplementary Table S1). To determine if the difference in embryo weight is responsible for the initial slow growth of *tcd1*, the weight of elite, *tcd1* and TCD1 embryos was determined by soaking seeds until fully imbibed (12 h), followed by dissecting the embryo from the endoplast. Isolated embryos were oven dried at 60 °C for two days then weighed. Due to the small size 20 isolated embryos were weighed together to calculate the average weight.

### 4.3. Role of Dhurrin on Sorghum Reproductive Fitness

To determine the effect of lack of dhurrin on sorghum reproductive fitness, elite, *tcd1* and TCD1 plants at the 3-leaf stage, were transferred to 250 mm pots with 8.5 L capacity and watered daily using a drip irrigation system. Plants were grown to maturity, and days to flower, final leaf number at flowering and total seed weight were recorded to analyse plant reproductive fitness.

### 4.4. Effect of GibberellicAacid (GA_3_) on Germination

To investigate the role of GA_3_ on seed germination and early growth, seeds were germinated and grown in vitro on vertical plates. Seeds of each genotype were surface sterilized with 6.25% sodium hypochlorite and 0.1% Tween-20 solution for 5 min followed by 30 s washing with 70% (*v*/*v*) ethanol. The final wash included 0.1% (*w*/*v*) mercuric chloride for 6 min after which seeds were rinsed in sterile double distilled water six times. After this seeds were placed in vertical square culturing plates (10 × 10 cm) containing half strength MS medium (2.22 g L^−1^ of MS Salts with Gamborg vitamins, 10 g L^−1^ of sucrose, 1.8% phytagel and pH adjusted to 5.7) [50] solidified with 1.8% phytagel. In the treatment group, 50 µM of GA_3_ (filter sterilised) was added to the medium after autoclaving, whereas the control treatment was just half strength MS medium. For each treatment there were three technical replicates for each of the five genotypes with each replicate having six biological replicates. Data for seed germination were recorded for 10 days after planting (DAP) the seeds on the medium. Plates were placed in a 28 °C constant temperature room under fluorescent light of approximately 100 µmol s^−1^ m^−2^ (16 h/day). Throughout the experiment pictures were taken every 6 h to create a 5 s time-lapse video of germinating seeds, for each vertical plate using Windows “Movie Maker 10” with frame rate of 29.97 s^−1^. The final germination percentage (FGP), mean germination time (MGT), germination index (GI) and median germination time (t50) were calculated by following Aravind et al. [51].

### 4.5. Role of Hormones in the Biosynthesis of Dhurrin

The potential role of plant hormones in dhurrin biosynthesis was investigated. All five genotypes elite, *acdc1*, ACDC1, *tcd1* and TCD1 were grown in 30-cell seedling trays under glasshouse conditions. A total of six different hormones consisting of salicylic acid (SA; 1 mM), indole-3-acetic acid (IAA; 100 µM), kinetin (KN; 20 µM), abscisic acid (ABA; 150 µM), methyl jasmonate (MeJa; 100 µM) and ethylene (E; 1 mM) with two controls (with and without bag) were used in this experiment. Due to the volatile nature of methyl jasmonate (MeJa) and ethylene (E), plants for both these treatments and their controls were covered with bags and kept in a separate glasshouse. Hormone solutions were sprayed 17 DAP, for the control the same carrier solution was sprayed without any hormone. To avoid the edge effect, plants from the middle of the seedling tray were chosen for harvest. To ensure that plants were at a similar ontogenetic stage individual with fully unfolded fourth leaf and height closest to the average for that particular genotype were selected for harvesting. Plants were harvested at 0, 2, 4, 8, 24, and 48 h after hormone application. Each treatment contained five genotypes with three replications per time-point. Tissue was oven dried, ground and used for cyanide assays.

### 4.6. Measurement of Cyanogenic Glucoside Concentrations

The dhurrin concentration was measured as cyanide potential (HCNp), i.e., the total amount of cyanide released from endogenous cyanogens using 9.5–10.5 mg of dried ground plant tissue following O’Donnell et al. [45]. Exogenous β-glucosidase (Sigma, Kawasaki, Japan, EC 3.2.1.21) in 0.1 M citrate buffer (trisodium citrate, Sigma), pH 5.6 was added in excess to ensure complete hydrolysis of dhurrin to HCN and the evolved HCN was captured in a 1 M NaOH solution and measured as NaCN in a colorimetric assay as described by Gleadow et al. [52]. Absorbance was measured at 580 nm using a microplate reader spectrophotometer (Fluostar Galaxy, BMG Labtechnologies). The cyanide concentrations were determined by comparison to the NaCN standard curve (0, 2, 5, 10, 20, 50, 100 and 150 µM) included in each microtitre plate and converted to the unit mg CN g^−1^ DW.

### 4.7. Data Measurements and Statistical Analysis

The seed emergence was recorded when at least 2 mm of shoot appeared from the soil. Seed germination was recorded when at least 2 mm of radical emerged from the seed. Stem height was measured from the base of the plant to the ligule of the youngest leaf. Qualitative data of plant stage were converted into numeric values using the ranking system given in Supplementary Table S1, prior to further data analysis. For the vertical plate experiment data for root and shoot length were measured from images using ImageJ software [53]. The significance of the data was calculated using analysis of variance (ANOVA) in IBM SPSS Statistics V25 [54]. In case of significant mean difference, *post hoc* comparisons were made using the Tukey’s test. For all tests *p* < 0.05 was considered significant.

## Figures and Tables

**Table 1:** Seedling and reproductive growth data of the selected three sorghum genotypes: Elite (parent line from which mutants are derived), *totally cyanide deficient class 1* (*tcd1*) mutant and sibling line of *tcd1* mutant without the target mutation (TCD1).

Genotype	Seed Mass (mg)	Embryo Mass (mg)	Flowering (DAP)	Leaf Number at Flowering	
Elite	18.0 ± 0.3 ^a^	0.26	91 ± 1 ^a^	12.4 ± 0.1 ^b^	
*tcd1*	19.8 ± 1.3 ^b^	0.31	103 ± 1 ^c^	12.4 ± 0.3 ^b^	
TCD1	20.1 ± 0.9 ^c^	0.42	97 ± 2 ^b^	11.9 ± 0.1 ^a^	

“DAP” = days after planting; Means with the same letter are not significantly different (*p* < 0.05) using Tukey’s HSD pairwise comparison between means.

## Supplementary Materials

The following are available online at https://www.mdpi.com/2223-7747/9/12/1791/s1, Figure S1. Movie of the germinating seedlings showing effect of gibberellic acid (GA3) on five sorghum genotypes 8 days after planting. Also via <https://youtu.be/fNWzvSFqchU>. Table S1. Conversion of qualitative plant developmental stage of Sorghum bicolor data into numeric data before data analysis.
