# Supplementary material for: Allocation of Resources to Cyanogenic Glucosides Does Not Incur a Growth Sacrifice in Sorghum bicolor (L.) Moench
Source: Plants (Basel). 2020 Dec 17;9(12):1791. doi: 10.3390/plants9121791 (PMC7766812; doi:10.3390/plants9121791)
Supplement: Supplementary file 1 [file plants-09-01791-s001.pdf]

Supplementary Information

Sohail, Blomstedt and Gleadow

Figure S1. A movie of the germinating seedlings can be found at following link here <https://youtu.be/fNWzvSFqchU>. Effect of gibberellic acid (GA3) on five sorghum genotypes 8 days after planting; A and B are tcd1 mutants; C and D are acdc1 mutants; E and F are TCD1 siblings; G and H are ACDC1 siblings; I and J are elite; A, C, E, G and I are control treatment; B, D, F, H and J are GA3 treatment.

Table S1. Conversion of qualitative plant developmental stage of *Sorghum bicolor* data into numeric data before data analysis.

Plant Stage	Number Assigned	Range
First leaf just emerged	1	(1)
First leaf is less than half unfolded	3	(2-4)
First leaf is more than half unfolded	7	(5-9)
First leaf is fully unfolded	11	(10-12)
Second leaf just emerged	13	(13)
Second leaf is less than half unfolded	15	(14-16)
Second leaf is more than half unfolded	19	(17-21)
Second leaf is fully unfolded	23	(22-24)
Third leaf just emerged	25	(25)
Third leaf is less than half unfolded	27	(26-28)
Third leaf is more than half unfolded	31	(29-33)
Third leaf is fully unfolded	35	(34-36)
Fourth leaf just emerged	37	(37)
Fourth leaf is less than half unfolded	39	(38-40)
Fourth leaf is more than half unfolded	43	(41-45)
Fourth leaf is fully unfolded	47	(46-48)
